# IgA-dominant post-infectious glomerulonephritis; making another case in support of renal biopsy in type 2 diabetic nephropathy

**DOI:** 10.15171/jrip.2016.10

**Published:** 2016-01-27

**Authors:** Ali Nayer, Gargi Davda, Rima Pai, Luis M. Ortega

**Affiliations:** ^1^Division of Nephrology and Hypertension, University of Miami Miller School of Medicine, Miami, FL, USA; ^2^Division of Nephrology and Hypertension, Allegheny Health Network, Temple University School of Medicine, Pittsburgh, PA, USA

**Keywords:** Infectious glomerulonephritis, Staphylococcal infection, Non-diabetic renal disease, Diabetic nephropathy

## Abstract

**Background:** Approximately one-third of individuals with type 2 diabetes mellitus will eventually develop diabetic nephropathy (DN). Impaired renal function in type 2 diabetics may also be secondary to non-diabetic renal disease (NDRD). NDRD in type 2 diabetics may occur alone in the absence of DN or may be superimposed on DN. Renal biopsy maybe indicated to establish the correct diagnosis and to ascertain the severity of glomerular and tubulointerstitial pathology.

**Case:** We report a patient with type 2 diabetes mellitus and chronic renal insufficiency who developed worsening of renal function in the setting of staphylococcal infection and antibiotic use.

**Conclusion:** Renal biopsy revealed IgA-dominant post-infectious glomerulonephritis and acute interstitial nephritis superimposed on diabetic glomerulosclerosis. Accumulating evidence indicates that, NDRD accounts for impaired renal function in a significant number of patients with type 2 diabetes mellitus. The presence of clinical, biochemical, and radiological features that suggest NDRD should prompt pathological evaluation of the kidney.

**Implication for health policy/practice/research/medical education:**

Non-diabetic renal disease may account for impaired renal function in a significant proportion of patients with type 2 diabetes mellitus. Thus, renal biopsy maybe necessary to elucidate the exact cause of renal disease in patients with type 2 diabetes. Histopathological evaluation of the kidney provides an accurate diagnosis and will guide treatment.

## Introduction

Diabetic nephropathy (DN) is a well-characterized microvascular complication of type 2 diabetes mellitus (T2DM). Approximately 20%-40% of individuals with T2DM will eventually develop type 2 diabetic nephropathy (T2DN). However, individuals with T2DM may also develop renal disease due to other causes. Non-diabetic renal disease (NDRD) may occur in individuals with T2DM in the absence of DN. Alternatively, NDRD may be superimposed on DN. T2DN is usually diagnosed based on the clinical, biochemical, and radiological grounds. However, there is great variation in the clinical presentation and biochemical findings. An individual with T2DM may require renal biopsy to determine the precise etiology of renal disease. Furthermore, renal biopsy provides invaluable information about the severity of glomerular and tubulointerstitial pathology in the kidney.

We report an elderly man with T2DM and chronic kidney disease who developed worsening of renal function in the setting of a systemic infection and antibiotic use. Renal biopsy revealed diabetic glomerulosclerosis, IgA-dominant post-infectious glomerulonephritis, and acute tubulointerstitial nephritis. We argue that renal biopsy should be considered in individuals with T2DM and renal disease.

## Case Presentation

**Figure 1:**
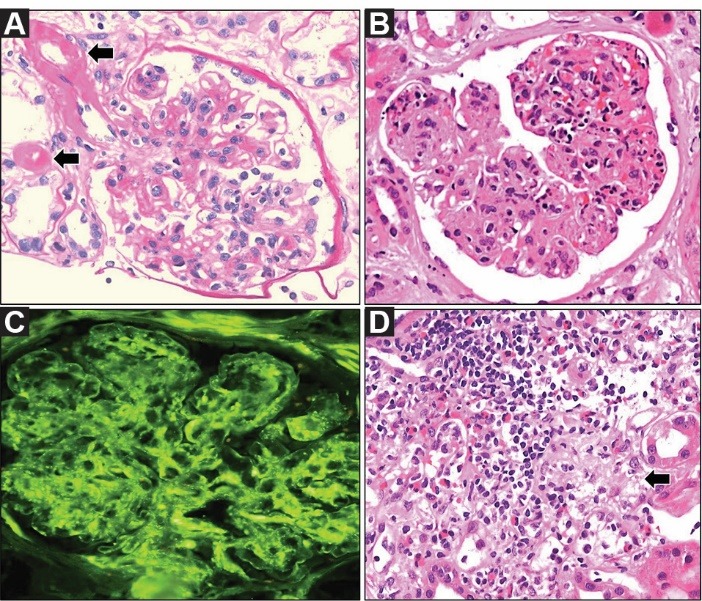
Pathologic findings of the kidney. (A) A glomerulus with thickening of capillary walls and mild mesangial expansion. Afferent and efferent arterioles showing hyalinosis (arrows) (periodic acid–Schiff stain); (B) A glomerulus demonstrating consolidation, hypercellularity, and polymorphonuclear leukocytes (hematoxylin and eosin stain); (C) Immunostaining showing glomerular IgA deposition (immunofluorescence microscopy); (D) Interstitium showing numerous mononuclear inflammatory cells and eosinophils. A poorly formed granuloma (arrow) is also seen (hematoxylin and eosin stain).

An 82-year-old Caucasian man with chronic kidney disease developed osteomyelitis and soft tissue infection of the left toe due to *Staphylococcus aureus*, for which piperacillin/tazobactam and vancomycin were administered. He was noted to have worsening renal function (serum creatinine: 5.9 mg/dl). Past medical history was notable for type 2 diabetes mellitus, chronic kidney disease (serum creatinine: 1.8 mg/dl), diabetic retinopathy and neuropathy, hypertension, and peripheral vascular disease. Hospital course was complicated by multilobar pneumonia, bacteremia, and purpura on upper extremities. Serological tests for vasculitis were negative. Transthoracic echocardiography showed no thrombi or valvular vegetations. Renal biopsy was performed. It showed diabetic glomerulosclerosis, diffuse proliferative glomerulonephritis, and tubulointerstitial nephritis with eosinophil and neutrophil aggregates as well as granulomas (Figure 1). Thirty-five percent of glomeruli were obsolescent. There was moderate to severe interstitial fibrosis and tubular atrophy. Afferent and efferent arterioles demonstrated hyalinosis. Immunofluorescence microscopy showed glomerular basement membrane staining for IgA (2+) and C3 (2+) (Figure 1). IgG, IgM, and C1q were negative. Electron microscopy showed focal subepithelial (hump-like) and subendothelial electron dense deposits in the glomerular basement membranes. The patient was diagnosed with IgA-dominant post-infectious glomerulonephritis and acute interstitial nephritis superimposed on DN. Worsening renal function led to initiation of hemodialysis. Prednisone taper was given to treat interstitial nephritis. Patient was discharged home after a prolonged hospital course. He presented with sepsis 3 months later while he was still requiring renal replacement therapy.

## Discussion

We report an elderly man with advanced T2DN who developed IgA-dominant post-infectious glomerulonephritis and acute tubulointerstitial nephritis associated with *Staphylococcus aureus* infection and antibiotic therapy.

Renal biopsy may be indicated in patients with T2DM and impaired kidney function to exclude concomitant or superimposed NDRD. Features that point to NDRD include signs and symptoms of a superimposed disorder such as autoimmunity or infections, sudden worsening of proteinuria (particularly if it is in the nephrotic range), hematuria more than a few red blood cells per high-power field, sterile pyuria, and faster-than-expected decline in renal function (1). There is considerable difference in the rate of decline in renal function between individuals with T2DM and renal disease. The rate of decline in glomerular filtration rate (GFR) was measured 5.2±4.1 (mean±SD) mL/min/year in 227 Caucasian T2DM patients with nephropathy over a 6.5-year follow-up period (2). Higher albuminuria, hemoglobin A1c, and systolic blood pressure, along with lower GFR and hemoglobin was associated with increased rate of decline in GFR. Therefore, a loss of GFR greater than 10 mL/min/year may indicate NDRD in Caucasian T2DM patients with nephropathy. Microscopic hematuria can occur in T2DN. In a study from Japan, microscopic hematuria was reported in 9.7% of biopsy-proven T2DN (3). Microscopic hematuria was associated with advanced diffuse glomerular and tubulointerstitial pathology as well as with higher serum creatinine level.

Several studies explored the prevalence of NDRD in T2DM patients with renal disease. Gonzalez Suarez and colleagues conducted a meta-analysis of 34 studies to explore the prevalence of NDRD in T2DM patients with renal disease (4). They found that T2DN was the only pathological finding in an average of 49.1% of renal biopsies. The prevalence of NDRD as the sole cause of renal disease in this cohort averaged 36.8%. An average of 14.1% of T2DM patients with renal disease demonstrated concurrent T2DN and NDRD. Considerable variation was noted in the prevalence of NDRD (14% and 83%) in type 2 diabetics with renal disease between studies. The prevalence of NDRD in T2DM was in part a function of race/ethnicity, geographic location, and the indications for renal biopsy.

A variety of NDRD can occur in diabetics including IgA nephropathy, membranous nephropathy, mesangial proliferative glomerulonephritis, hypertensive renal disease, minimal change disease, and focal segmental glomerulosclerosis (4-6). In a retrospective study from the United States, Sharma and colleagues studied renal biopsies from diabetic patients, the vast majority of whom (97.4%) had T2DM (5). The prevalence of DN, NDRD, and DN+NDRD was 37%, 36%, and 27%, respectively. The most frequent causes of NDRD included focal segmental glomerulosclerosis (22%), hypertensive nephrosclerosis (18%), acute tubular necrosis (17%), IgA nephropathy (11%), membranous nephropathy (8%), and pauci-immune glomerulonephritis (7%). The leading causes of renal disease in the DN+NDRD group were acute tubular necrosis (43%), hypertensive nephrosclerosis (19%), focal segmental glomerulosclerosis (13%), and IgA nephropathy (7%). This study showed that NDRD alone or superimposed on DN was the most common histopathological diagnosis in diabetic patients who underwent renal biopsy. In a study from China, Zhuo and colleagues examined 216 renal biopsies from individuals with T2DM and renal disease (6). The vast majority of biopsies showed NDRD (82.9 %) alone. Concurrent T2DN and NDRD was diagnosed in 10.7 % of biopsies. The prevalence of T2DN alone in this cohort was only 6.5 %. While IgA nephropathy was the leading cause of renal disease in approximately one-third of individuals 60 years of age or younger, membranous nephropathy and tubulointerstitial disease predominated in individuals older than 60 years.

The absence of diabetic retinopathy does not exclude the possibility of DN in T2DM patients with impaired renal function. In a study from Denmark, diabetic retinopathy was present in only 56% of T2DM patients with biopsy-proven T2DN (7). Similarly, up to 47.5% of the hypertensive T2DM patients with overt proteinuria in Germany had no evidence of diabetic retinopathy (8). Moreover, approximately 40% of dialysis-dependent T2DM patients showed no evidence of diabetic retinopathy.

IgA-dominant acute postinfectious glomerulonephritis (APIGN) is a variant of acute postinfectious glomerulonephritis that is commonly associated with staphylococcal infections (9). APIGN is frequently associated with cutaneous infections in older diabetics. APIGN manifests as proteinuria, hematuria, acute renal insufficiency, and hypocomplementemia. Immunofluorescence microscopy reveals glomerular deposition of IgA and C3. APIGN has a poor renal prognosis. The majority of patients (84%) sustain impaired renal function or progress to end-stage renal disease. Our elderly patient developed APIGN associated with *Staphylococcus aureus* infection superimposed on T2DN.

## Conclusion

In summary, accumulating evidence shows a high prevalence of NDRD in type 2 diabetics with renal disease. The presence of atypical clinical and biochemical features should prompt pathological evaluation of the kidney.

## Authors’ contribution

AN, GD, RP, and LMO prepared the manuscript equally.

## Conflicts of interest

The authors declare that they have no conflicting interest.

## Ethical considerations

Ethical issues (including plagiarism, data fabrication, double publication) have been completely observed by the authors.

## Funding/Support

None.
